# *Radix Bupleuri Chinensis* Extract Ameliorates Doxorubicin-Induced Spermatogenic Dysfunction: Involvement of the GnRHR Signaling Pathway and Taurine Biosynthesis

**DOI:** 10.3390/ph19081280

**Published:** 2026-08-13

**Authors:** Manyu Wang, Yang Fu, Peipei Yuan, Yi Zeng, Zihao Yang, Shenggui Zhang, Weisheng Feng, Xiaoke Zheng

**Affiliations:** 1 Department of Pharmacy, Henan University of Chinese Medicine, Zhengzhou 450046, China; wang14552023@163.com (M.W.); 15136153630@163.com (P.Y.); zengyi38875749@163.com (Y.Z.); yang1213666@163.com (Z.Y.); m18203873057_1@163.com (S.Z.); fwsh@hactcm.edu.cn (W.F.); 2 The Engineering and Technology Center for Chinese Medicine Development of Henan Province, Zhengzhou 450046, China

**Keywords:** *Radix Bupleuri Chinensis* extract, doxorubicin, spermatogenic dysfunction, GnRH signaling pathway, taurine, meiosis

## Abstract

**Background**: Male infertility, largely driven by declining sperm quality, is a growing global concern. *Radix Bupleuri Chinensis* (Chaihu, CH)—a key component of traditional formulas such as Chaihu Shengjing Tang and Chaihu Shugan San—has been clinically used to enhance sperm quality. However, the specific role and mechanism of CH as a single entity in ameliorating spermatogenic dysfunction remain unclear. This study aimed to determine whether CH extract ameliorates spermatogenic dysfunction and whether GnRH/GnRHR signaling and taurine biosynthesis are involved. **Methods**: Male Kunming (KM) mice and GC-1 spermatogonial cells were used to establish experimental models. Histopathological and cellular changes were examined via hematoxylin and eosin (H&E) staining and immunofluorescence. Enzyme and hormone levels were quantified, while gene and protein expression were analyzed using quantitative real-time polymerase chain reaction, Western blotting, and high-content imaging. **Results**: CH extract mitigated DOX-induced spermatogenic dysfunction by activating the GnRHR signaling pathway, enhancing taurine biosynthesis, and reducing oxidative stress. In vitro, saikosaponin D alleviated DOX-induced injury in GC-1 cells and was associated with changes in GnRHR-related signaling and taurine-biosynthesis-related proteins, supporting its potential role as a candidate bioactive constituent of CH. **Conclusions**: CH extract alleviated DOX-induced spermatogenic impairment in mice. The in vivo cetrorelix experiments support the involvement of the GnRHR signaling pathway, whereas the concurrent changes in taurine, CDO1, and FMO1 are consistent with enhanced taurine biosynthesis. These findings provide experimental support for further investigation of CH as a potential intervention for chemotherapy-related reproductive injury.

## 1. Introduction

Doxorubicin (DOX), an anthracycline antibiotic, is a widely used cytotoxic chemotherapeutic agent in clinical oncology [1]. It is routinely administered to treat various malignancies in adults and adolescents, including leukemia, lymphoma, and bladder cancer [2,3,4]. DOX exerts its antineoplastic activity by targeting rapidly proliferating cells, inducing chromosomal aberrations in spermatogonia, arresting the cell cycle, and triggering spermatogonial apoptosis, thereby disrupting spermatogenesis and contributing to male infertility [5,6].

Traditional Chinese Medicine (TCM), a cornerstone of Chinese civilization, has been extensively applied in treating male infertility [7]. Clinical studies demonstrate that liver-soothing and kidney-tonifying formulas—such as Chaihu Shengjing Tang (Bupleurum Sperm-Producing Decoction) and Chaihu Shugan San (Bupleurum Liver-Coursing Powder)—can enhance sperm density, motility, and survival rate, thereby improving conception outcomes [8,9]. Notably, Chaihu Shugan San and related formulas have been widely reported in the management of male reproductive disorders [10]. In the treatment of infertility, modified Chaihu Shugan San has been shown to significantly improve sperm quality in patients with asthenospermia [9,11,12]. Specifically, one study reported that the liver-soothing and depression-relieving approach significantly enhanced semen parameters in asthenospermia patients [13], while another demonstrated the efficacy of syndrome-based differentiation in treating asthenospermia with Chaihu Shugan San [11]. Beyond sperm quality improvement, Chaihu Shugan San has also shown therapeutic potential in related conditions such as category IIIA prostatitis complicated by semen liquefaction disorders [14], further supporting its role in male reproductive healthcare. Data mining studies of renowned TCM practitioners have further confirmed that CH is among the core herbs frequently used in male infertility treatment [15]. These clinical findings collectively underscore the therapeutic potential of CH in mitigating testicular injury and improving male reproductive function.

Beyond fertility treatment, CH-containing formulas have been extensively documented in the treatment of various male reproductive disorders. Early clinical observations reported that Xiaochaihu plus Shigao Tang (Minor Bupleurum plus Gypsum Decoction) showed effectiveness in treating orchitis secondary to mumps [16]. Subsequently, modified Chaihu Shugan San was successfully used to alleviate testicular pain and swelling in case reports [17], and later demonstrated remarkable efficacy in a larger study of 37 orchitis patients, achieving a total effective rate of 97.3% with 32 cases fully recovered [18]. CH-containing formulas have also been reported to relieve testicular pain, with one study documenting 32 cases successfully treated with modified Chaihu Guizhi Tang (Bupleurum and Cinnamon Twig Decoction) [19]. More recently, for epididymitis, modified Xiaochaihu Tang (Minor Bupleurum Decoction) has demonstrated significant clinical efficacy [20], particularly in cases of acute epididymitis with encapsulated abscess [21]. These clinical observations, spanning over three decades, collectively highlight the broad and enduring applicability of CH-containing formulas in male reproductive healthcare.

Building on these clinical findings, experimental studies have begun to elucidate the underlying mechanisms. CH-containing formulas have been shown to protect against reproductive toxicity, as demonstrated by the antagonistic effect of Xiaochaihu Tang against dichlorvos-induced reproductive damage [22]. Chaihu Shugan Tang has been found to ameliorate erectile dysfunction in rats with liver depression syndrome [23,24]. These mechanistic insights provide a scientific basis for the clinical efficacy of CH-containing formulas.

CH is known as an essential medicinal herb for soothing the liver and relieving stagnation. It serves as the monarch drug in most clinically commonly used formulas for soothing the liver and regulating *qi*. Its traditional medicinal effects mainly include soothing the liver and relieving stagnation, dispelling heat and relieving exterior syndrome, and lifting yang *qi* [25]. However, while the presence of CH in these multi-herb formulations suggests its relevance to male reproductive health, the therapeutic effects of complex formulas cannot be directly attributed to any single constituent without systematic investigation. To date, whether CH alone exerts direct protective effects on spermatogenesis—and through which molecular pathways—remains largely unexplored. Therefore, although the traditional use of CH-containing formulas provides a compelling ethnopharmacological foundation, the specific action of CH itself and its underlying mechanisms represent a significant knowledge gap that the present study aims to address.

Preliminary findings from our laboratory indicate that CH extract increases the testicular index, enhances gonadotropin-releasing hormone (GnRH) secretion in rats, and upregulates testicular GnRH receptor (GnRHR) expression—collectively suggesting its potential role in supporting testicular function [26]. Activation of the GnRHR signaling pathway markedly attenuates DOX-induced spermatogenic disruption [27,28]. Accordingly, this study aims to elucidate the biological mechanism through which CH ameliorates DOX-induced spermatogenic dysfunction, providing insight into potential strategies for mitigating the reproductive side effects of anticancer therapies.

## 2. Results

### 2.1. CH Protects Against DOX-Induced Testicular Dysfunction in Mice

**Figure 1:**
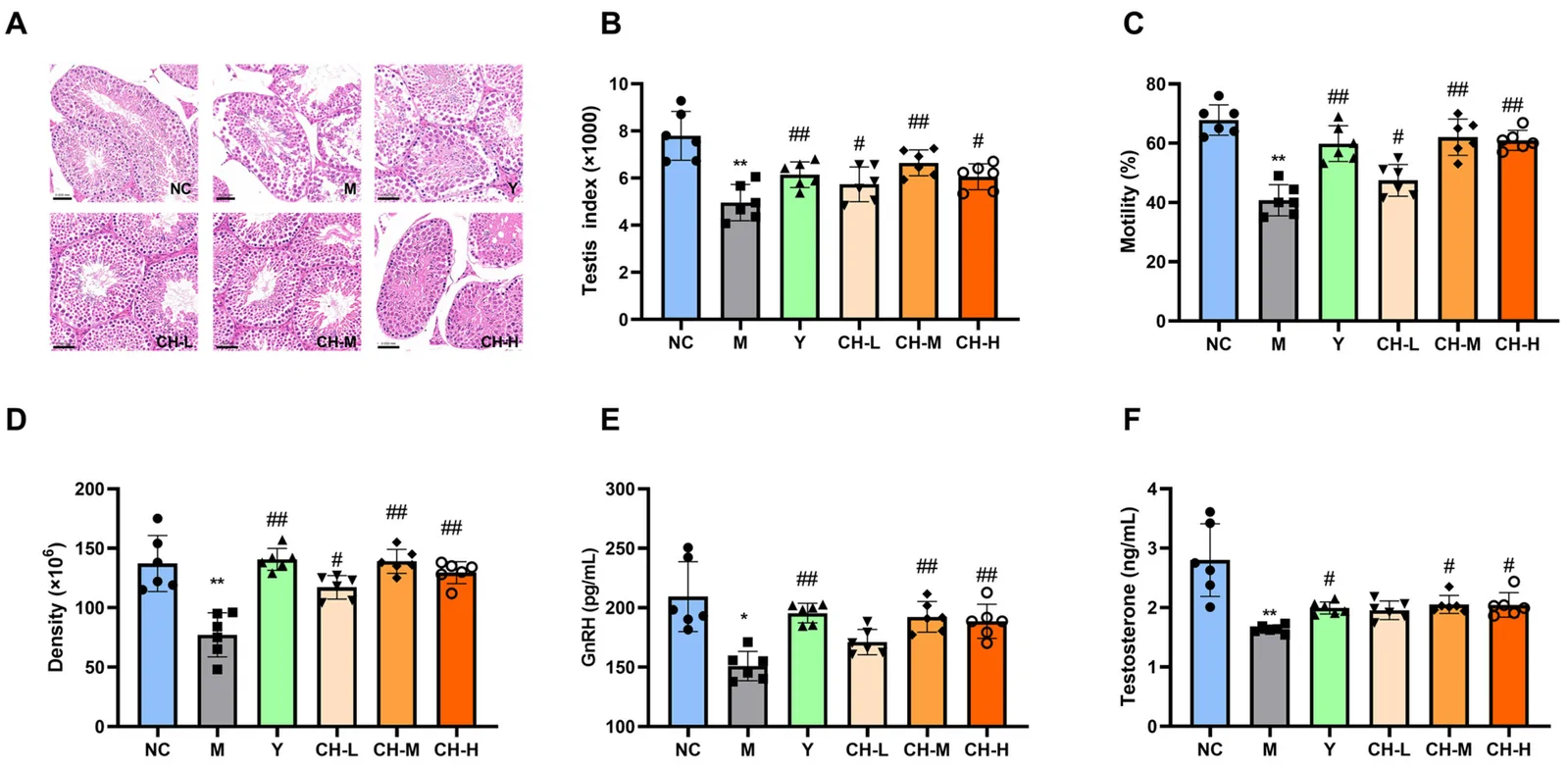
CH confers protection against DOX-induced testicular dysfunction in mice. (**A**) Representative H&E-stained sections of mouse testicular tissue across experimental groups. Scale bar = 50 μm. (**B**) Testicular index of mice in different treatment groups. (**C**,**D**) Sperm motility and density analyses in experimental mice. (**E**,**F**) Serum concentrations of gonadotropin-releasing hormone (GnRH) and testosterone in the treatment groups. Data are presented as mean ± SD; * *p* < 0.05, ** *p* < 0.01 vs. NC group; ^#^ *p* < 0.05, ^##^ *p* < 0.01 vs. M group.

H&E staining (Figure 1A) showed disorganization of the germ-cell layers and fewer spermatozoa within the seminiferous tubules after DOX treatment. Compared with the control group, DOX-treated mice had lower testicular indices, sperm motility, and sperm density (Figure 1B–D). Serum GnRH and testosterone levels were also reduced (*p* < 0.01; Figure 1E,F). CH-M and CH-H partially restored these parameters. No significant changes in the measured hepatic or renal function indices were detected in the CH-H group (Supplementary Figure S1).

### 2.2. Effects of CH on GnRHR and Meiosis-Related Indicators in the Testis of DOX-Treated Mice

**Figure 2:**
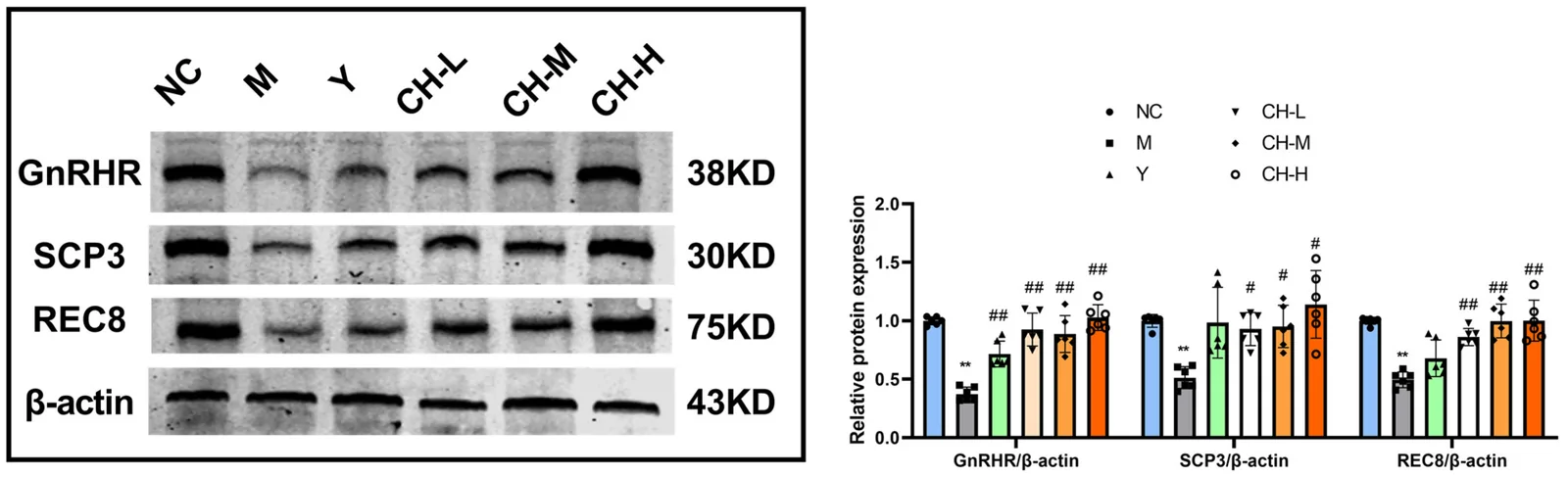
Effects of CH on GnRHR and meiosis-related indicators in the testis of DOX-treated mice. Western blot analysis of GnRHR, SCP3, and REC8 expression in the testicular tissues of DOX-treated mice. Data are presented as mean ± SD. from three independent experiments. ** *p* < 0.01 vs. NC group; ^#^ *p* < 0.05, ^##^ *p* < 0.01 vs. M group.

To evaluate the regulatory effects of CH on GnRHR and meiosis-related proteins, Western blot (WB) analysis was performed. WB showed that DOX reduced testicular GnRHR, SCP3, and REC8 protein levels, whereas CH treatment partially restored their expression (Figure 2). These changes were associated with improved spermatogenic indices.

### 2.3. Effects of CH on Improving Spermatogenic Dysfunction Are Closely Related to the GnRH Signaling Pathway

**Figure 3:**
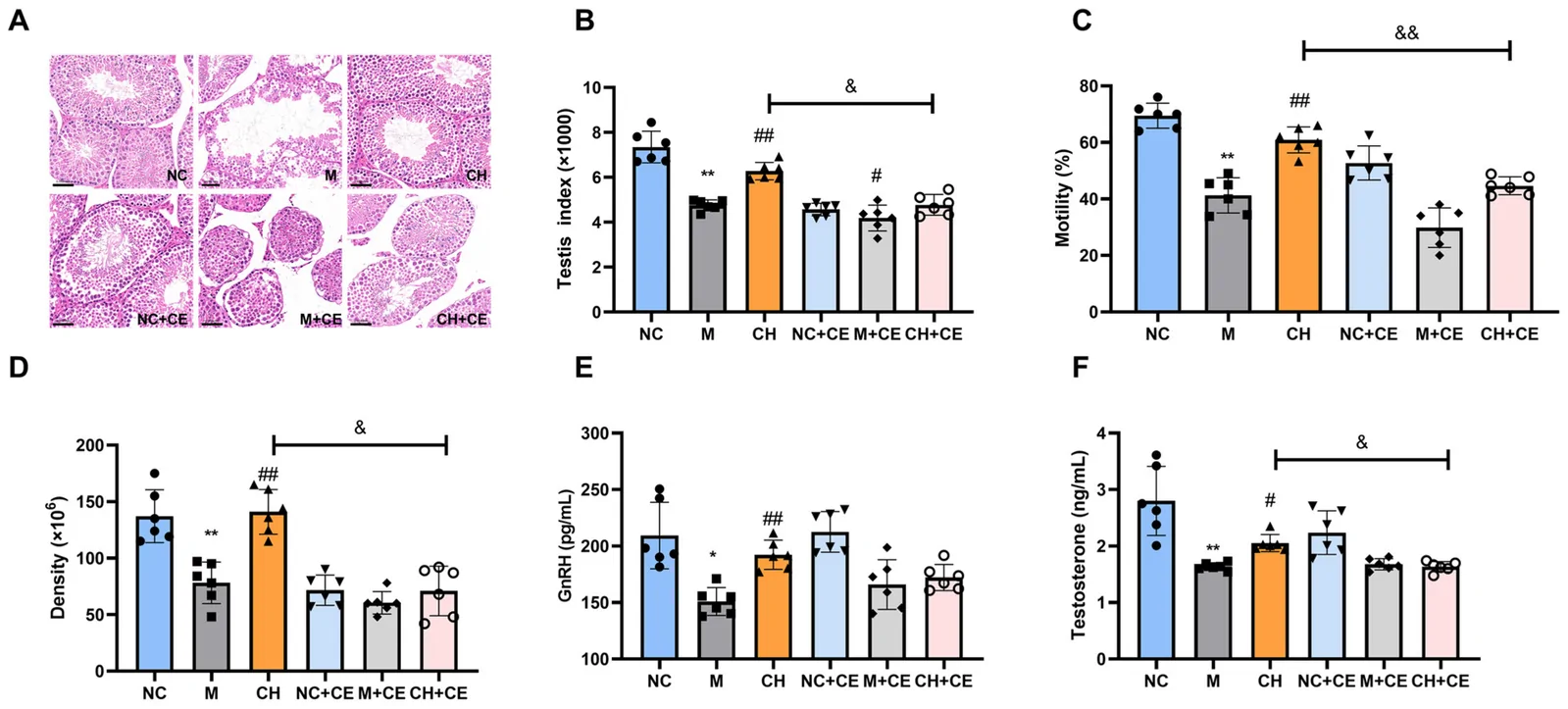
Effects of CH on improving spermatogenic dysfunction are closely related to the GnRH signaling pathway. (**A**) Representative H&E-stained sections of mouse testicular tissues across the experimental groups. Scale bar = 50 μm. (**B**) Testicular index of mice in different treatment groups. (**C**,**D**) Sperm motility and density analyses in experimental mice. (**E**,**F**) Serum concentrations of gonadotropin-releasing hormone (GnRH) and testosterone in the treatment groups. After administering cetrorelix, the ameliorative effect of CH on DOX-induced spermatogenic dysfunction was attenuated. Data are presented as mean ± SD; * *p* < 0.05 and ** *p* < 0.01 versus the NC group; ^#^ *p* < 0.05 and ^##^ *p* < 0.01 versus the M group; and *p* < 0.05 and ^&&^ *p* < 0.01 versus the CH group, ^&^ indicates the difference between CH + CE and CH groups.

The medium concentration of CH was selected for the subsequent experiments. Further validation was performed using a GnRH antagonist. The results showed that administration of the GnRH antagonist cetrorelix attenuated the beneficial effects of CH on spermatogenic parameters (Figure 3A). Specifically, CH-induced improvements in testicular index, sperm motility, sperm density, GnRH and testosterone production were significantly attenuated (Figure 3B–F), further confirming that CH’s protective effects support the involvement of GnRH signaling.

### 2.4. CH Counters Testicular Oxidative Stress Through the GnRH Signaling Pathway

**Figure 4:**
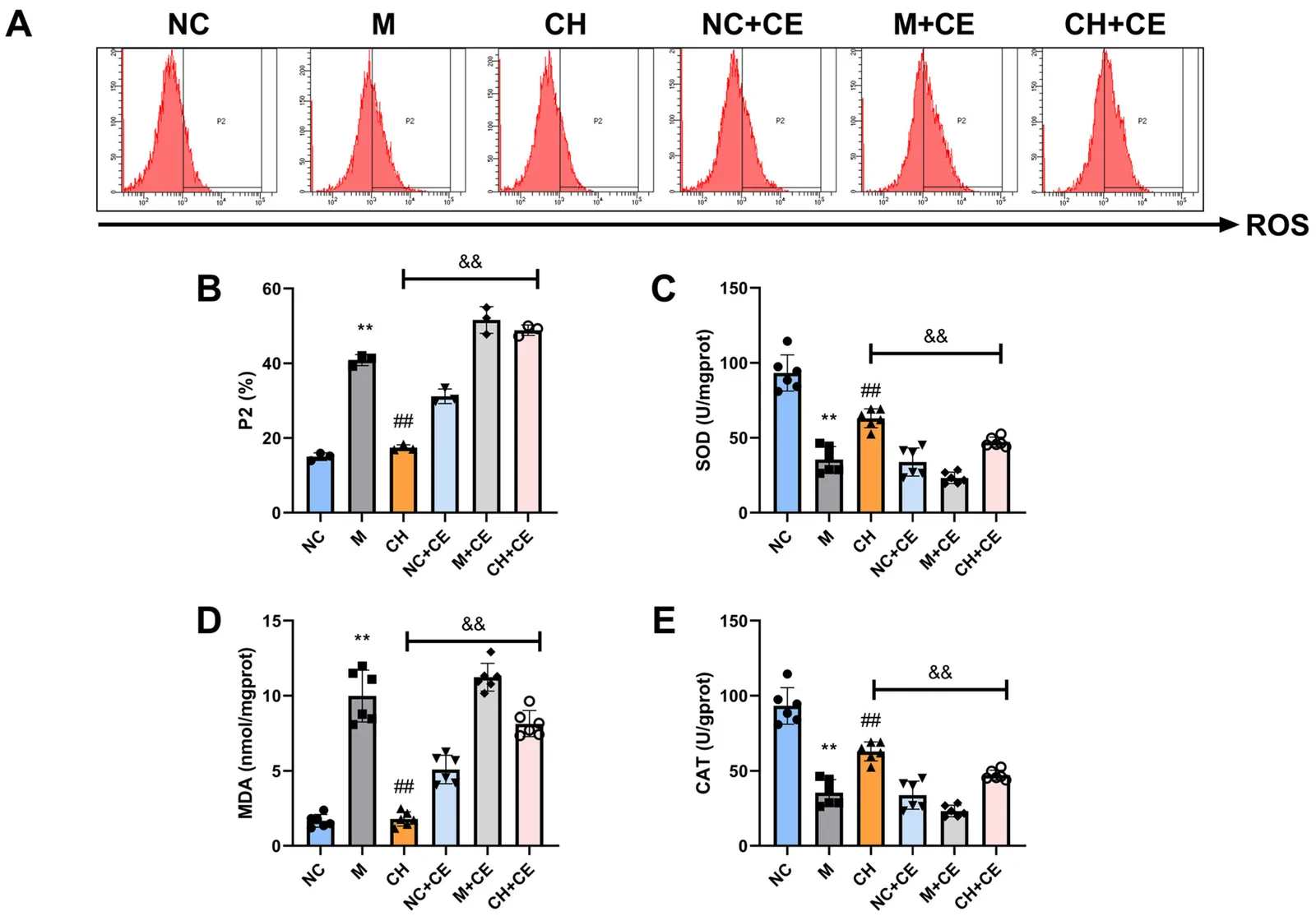
CH counters testicular oxidative stress through the GnRH signaling pathway. The levels of (**A**,**B**) ROS, (**C**) SOD, (**D**) MDA, and (**E**) CAT in the testicular tissues of mice in each group. Data are presented as mean ± SD; ** *p* < 0.01 vs. NC group; ^##^ *p* < 0.01 vs. M group; ^&&^ *p* < 0.01 vs. the CH group.

To evaluate the antioxidant effect of CH, we detected oxidative stress indicators. The results showed that DOX treatment markedly elevated reactive oxygen species (ROS) and malondialdehyde (MDA) levels while suppressing superoxide dismutase (SOD) and catalase (CAT) activities in testicular tissue. In contrast, CH administration significantly reduced ROS and MDA levels and markedly increased SOD and CAT activities in the testes (Figure 4). However, co-administration of the GnRH antagonist cetrorelix attenuated these protective effects, indicating that CH-mediated attenuation of testicular oxidative stress supports the involvement of GnRH signaling.

### 2.5. Cetrorelix Attenuates CH-Associated Changes in Taurine-Related and Meiosis-Related Markers

**Figure 5:**
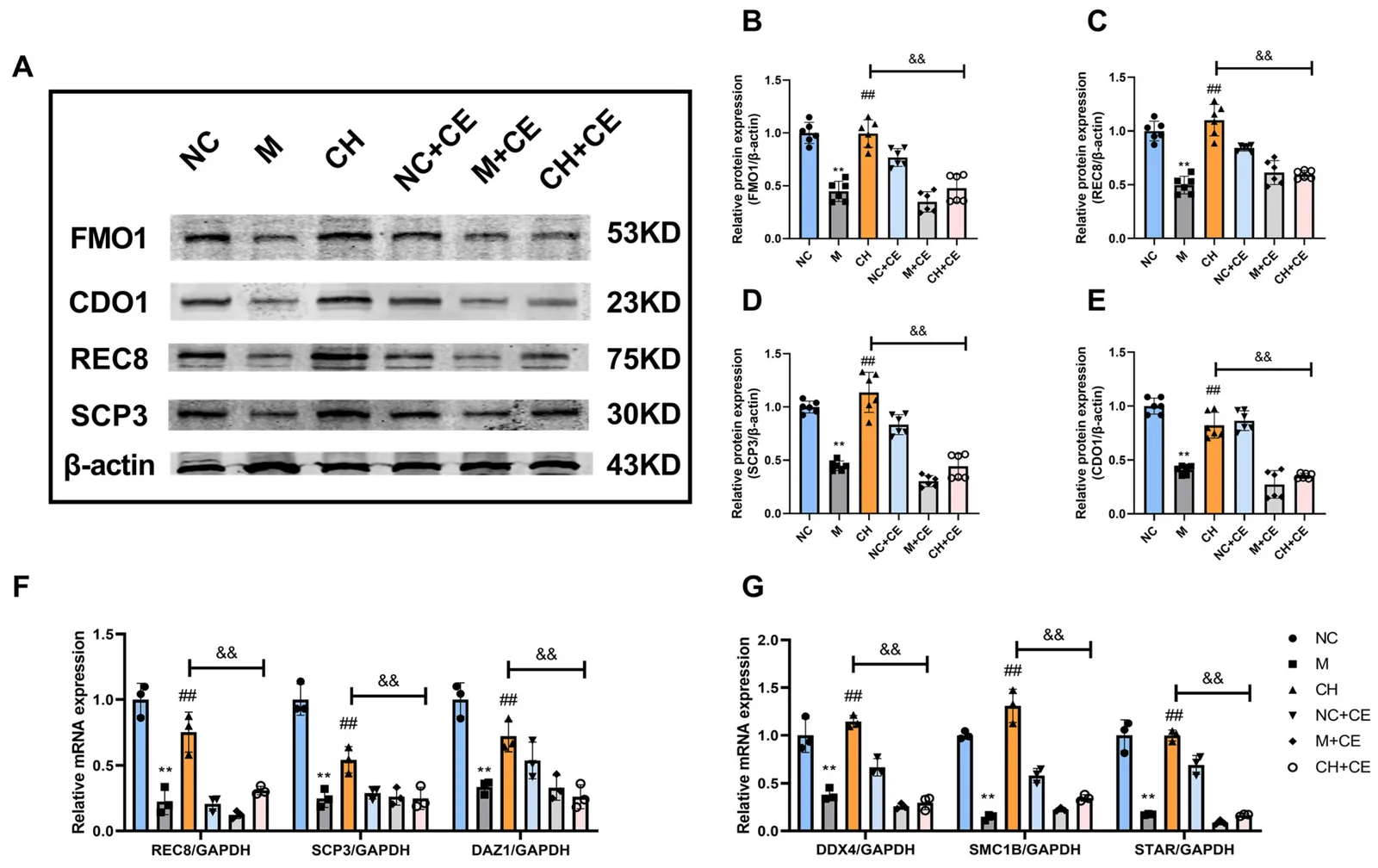

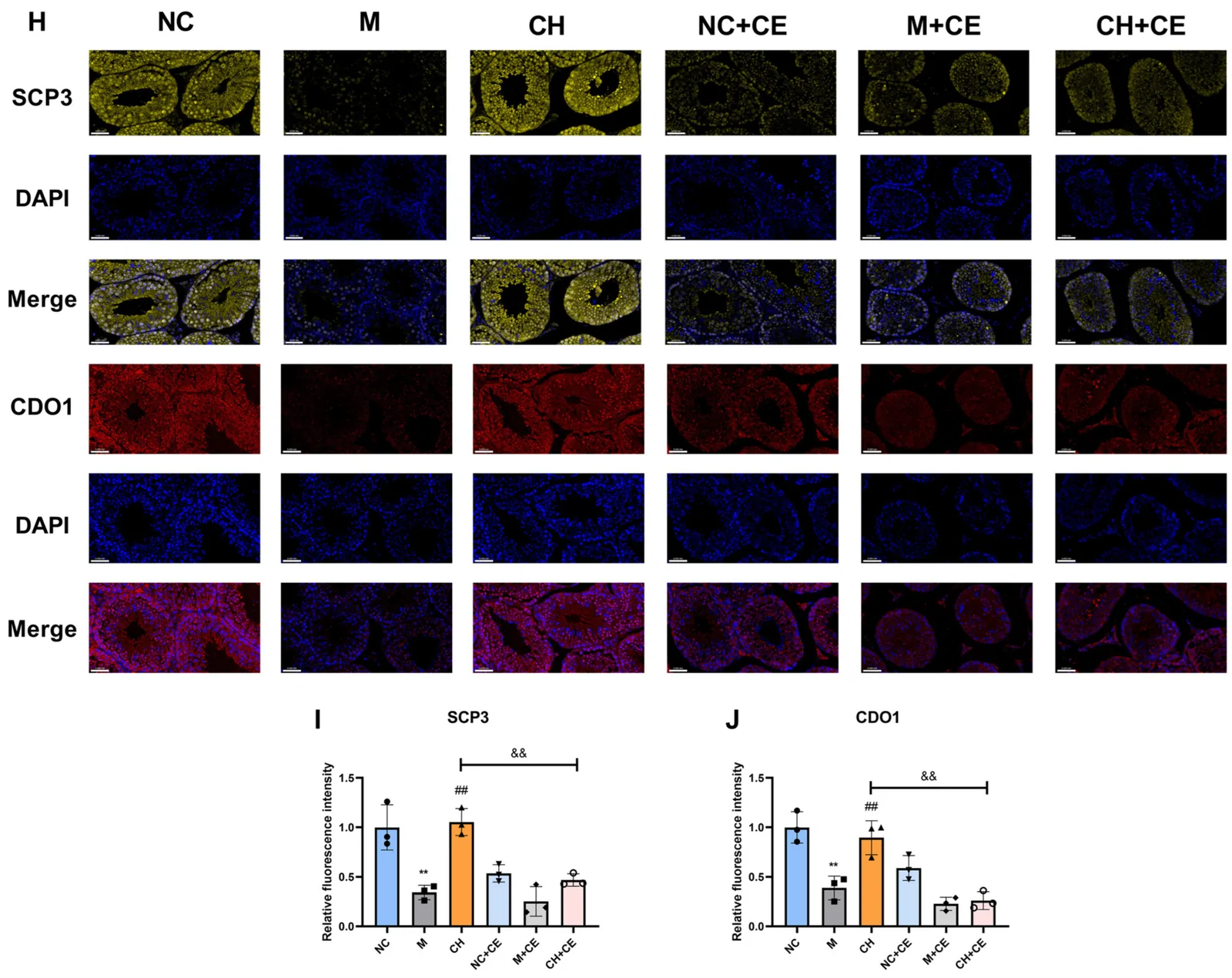
GnRHR signaling critically mediates CH’s regulation of taurine synthesis and meiotic proteins. (**A**–**E**) WB analysis for FMO1, CDO1, REC8 and SCP3 protein expression in testicular tissues from DOX-treated mice. (**F**,**G**) qRT-PCR analysis of meiotic gene expression (REC8, SCP3, DAZ1, DDX4, and SMC1B) and steroidogenic acute regulatory protein (STAR) mRNA levels in testicular tissues from DOX-treated mice. (**H**–**J**) Immunofluorescence (IF) detection of the SCP3 and CDO1 proteins in mouse testicular sections. Scale bar = 50 μm. Data are presented as mean ± SD. ** *p* < 0.01 vs. NC group; ^##^ *p* < 0.01 vs. M group; ^&&^ *p* < 0.01 vs. the CH group.

Western blot results showed that CH markedly enhanced the testicular expression of taurine-synthesizing enzymes CDO1 and FMO1, as well as the meiotic proteins REC8 and SCP3 (Figure 5A–E). These stimulatory effects were entirely negated by cetrorelix treatment. Consistent with these findings, qPCR analysis revealed that CH significantly upregulated the expression of key meiotic genes (REC8, SCP3, DAZ1, DDX4, SMC1B, and STAR), whereas GnRH antagonism abolished these transcriptional effects (Figure 5F,G). Immunofluorescence analysis further confirmed the localization and expression patterns of SCP3 and CDO1, corroborating the Western blot results (Figure 5H–J).

Collectively, these data show that CH-associated changes in taurine-related and meiosis-related markers were sensitive to cetrorelix.

### 2.6. CH Ameliorates Spermatogenic Dysfunction Through the GnRHR Signaling Pathway

**Figure 6:**
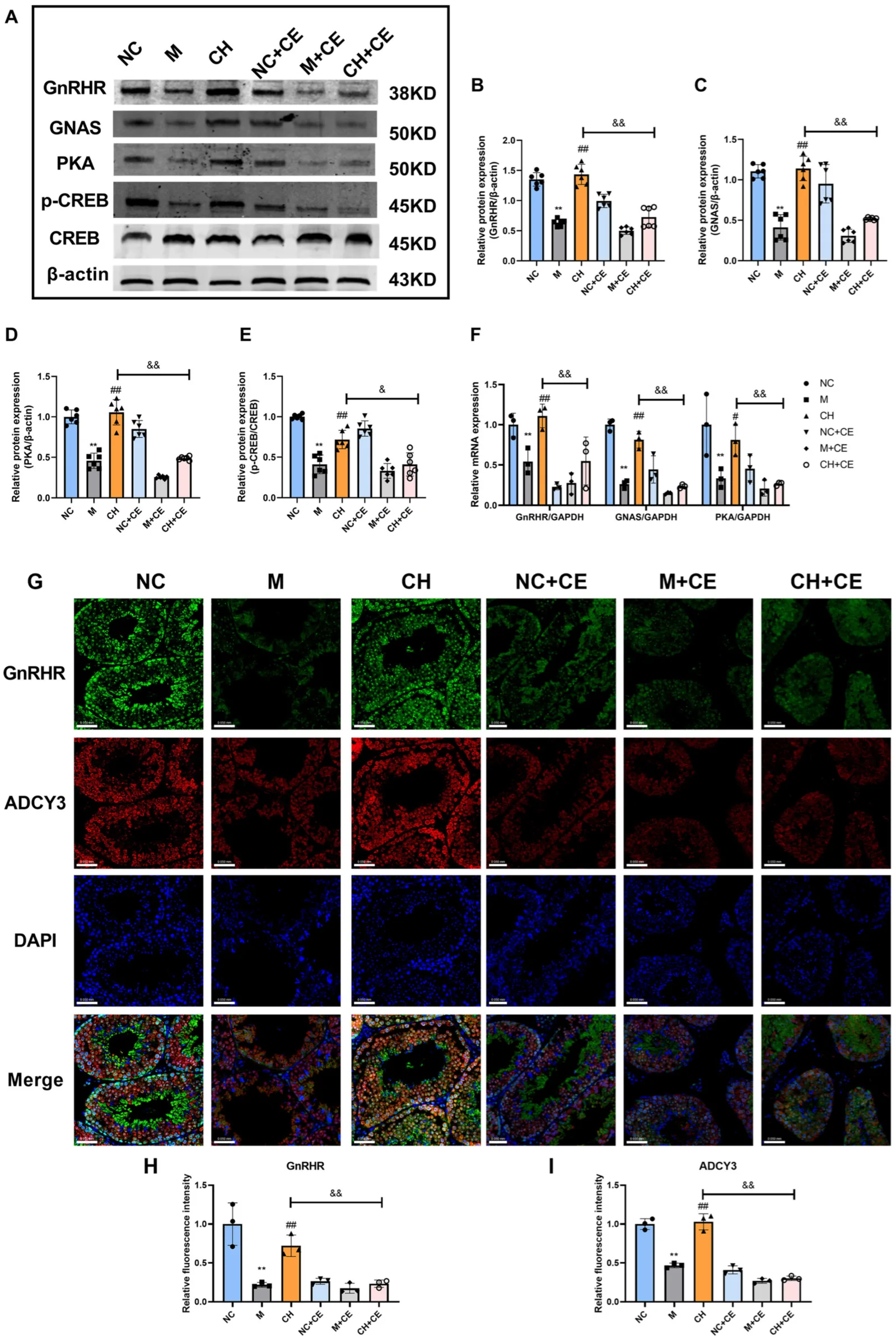
CH ameliorates spermatogenic dysfunction through the GnRHR signaling pathway. (**A**–**E**) Western blot analysis of the protein expression of GnRHR, GNAS, PKA, and p-CREB/CREB in testicular tissues from DOX-treated mice. (**F**) qRT-PCR analysis for *GnRHR*, *GNAS*, and *PKA* expression in testicular tissues from DOX-treated mice. (**G**–**I**) Immunofluorescence (IF) detection of the GnRHR and ADCY3 proteins in mouse testicular sections. Scale bar = 50 μm. Data are presented as mean ± SD. ** *p* < 0.01 vs. NC group; ^#^ *p* < 0.05, ^##^ *p* < 0.01 vs. M group; ^&&^ *p* < 0.01 vs. the CH group, ^&^ indicates the difference between CH + CE and CH groups.

We hypothesized that CH mitigates DOX-induced spermatogenic dysfunction via activation of the GnRHR signaling pathway in the testes. To test this, we evaluated the expression of GnRHR, GNAS, PKA, and the phosphorylation ratio of p-CREB/CREB. CH significantly upregulated GnRHR expression and increased GNAS, PKA, and phosphorylation levels of CREB (Figure 6A–E), whereas these effects were completely abolished by cetrorelix. The qPCR analysis further confirmed that CH markedly elevated mRNA expression of GnRHR, GNAS, and PKA (Figure 6F). Immunofluorescence analysis revealed increased expression of GnRHR and ADCY3 proteins following CH treatment, which was markedly attenuated by GnRHR antagonism (Figure 6G–I). These findings indicate that CH ameliorates DOX-induced spermatogenic dysfunction via the GnRHR signaling pathway.

### 2.7. SSD Exerts Protective Effects Against GC-1 Cells Injured by DOX

**Figure 7:**
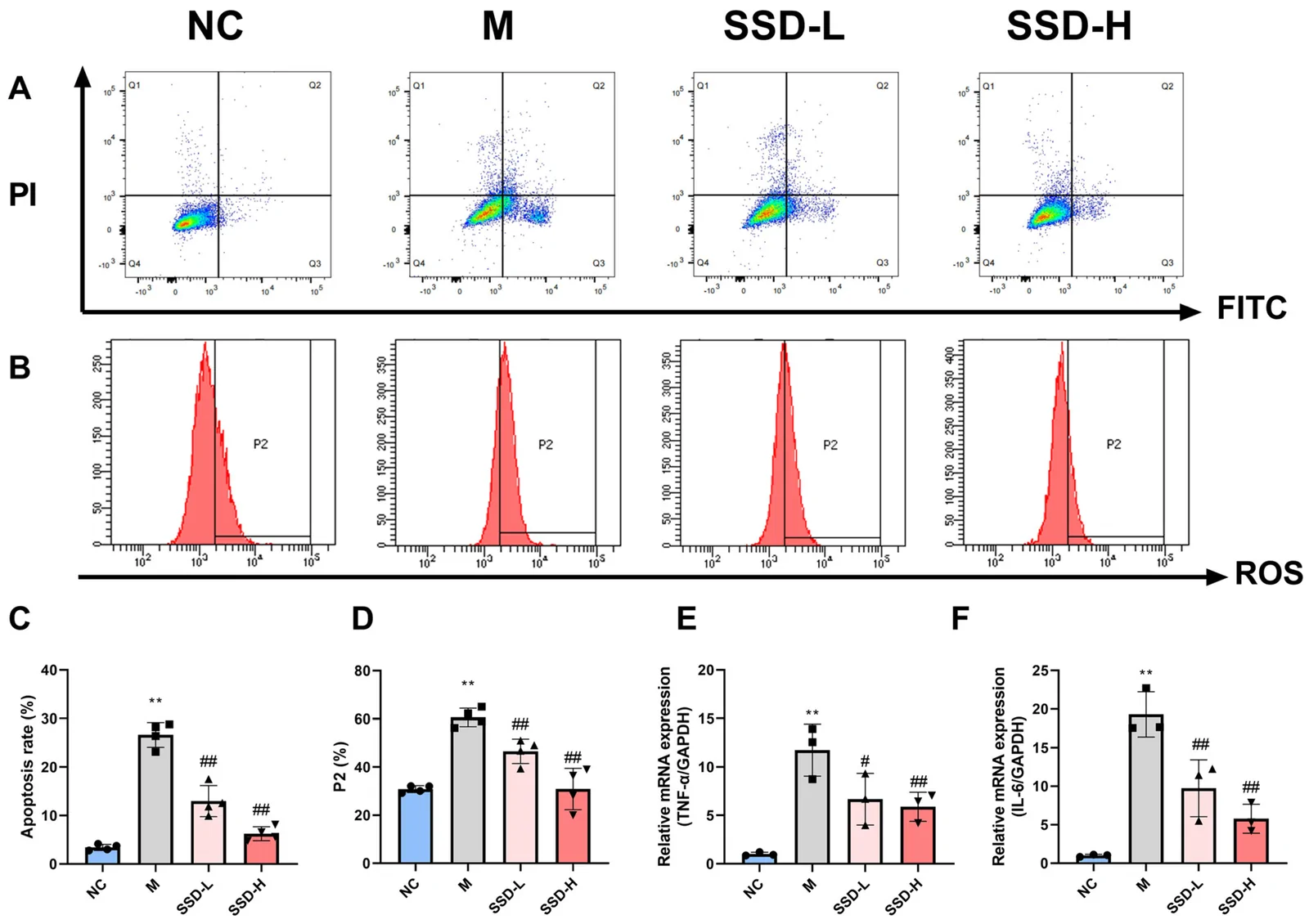
SSD attenuates DOX−induced injury in GC-1 cells. (**A**–**D**) Apoptosis and ROS levels. (**E**,**F**) *TNF-α* and *IL-6* mRNA levels in GC-1 cells. Data are presented as mean ± SD. from three independent experiments. ** *p* < 0.01 vs. NC group; ^#^ *p* < 0.05, ^##^ *p* < 0.01 vs. M group.

LC–MS analysis detected saikosaponins A and D in the CH extract (Supplementary Figure S2 and Table S1). CCK-8 assays showed that saikosaponin D (SSD) reduced DOX-induced cytotoxicity in GC-1 cells (Supplementary Figure S5). SSD was therefore selected as a candidate bioactive constituent for further evaluation. Both SSD concentrations reduced apoptosis and ROS accumulation (Figure 7A–D) and lowered *TNF-α* and *IL-6* mRNA levels (Figure 7E,F).

### 2.8. SSD Is Associated with Changes in GnRHR-Related and Taurine-Biosynthesis-Related Markers in GC-1 Cells

**Figure 8:**
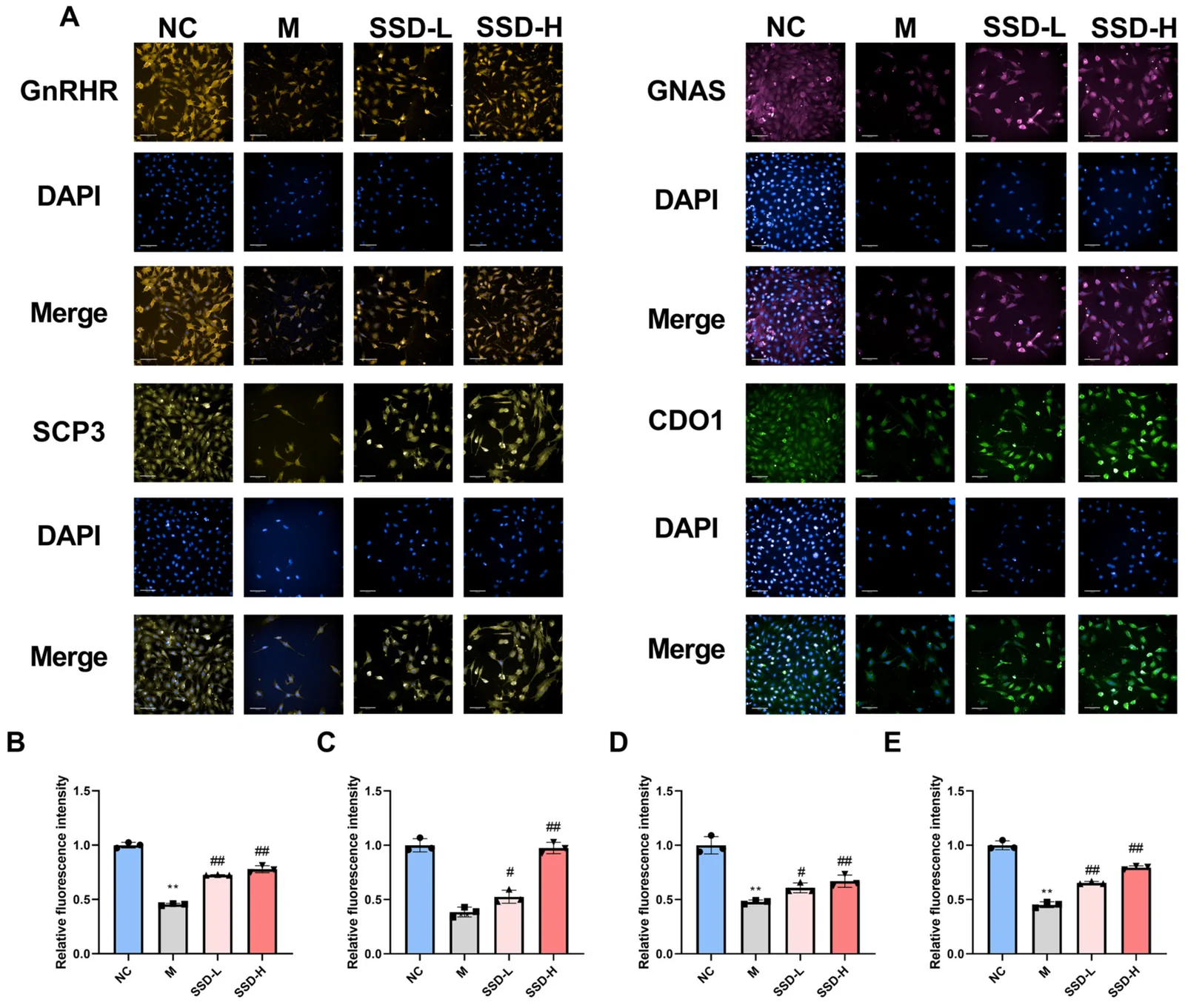

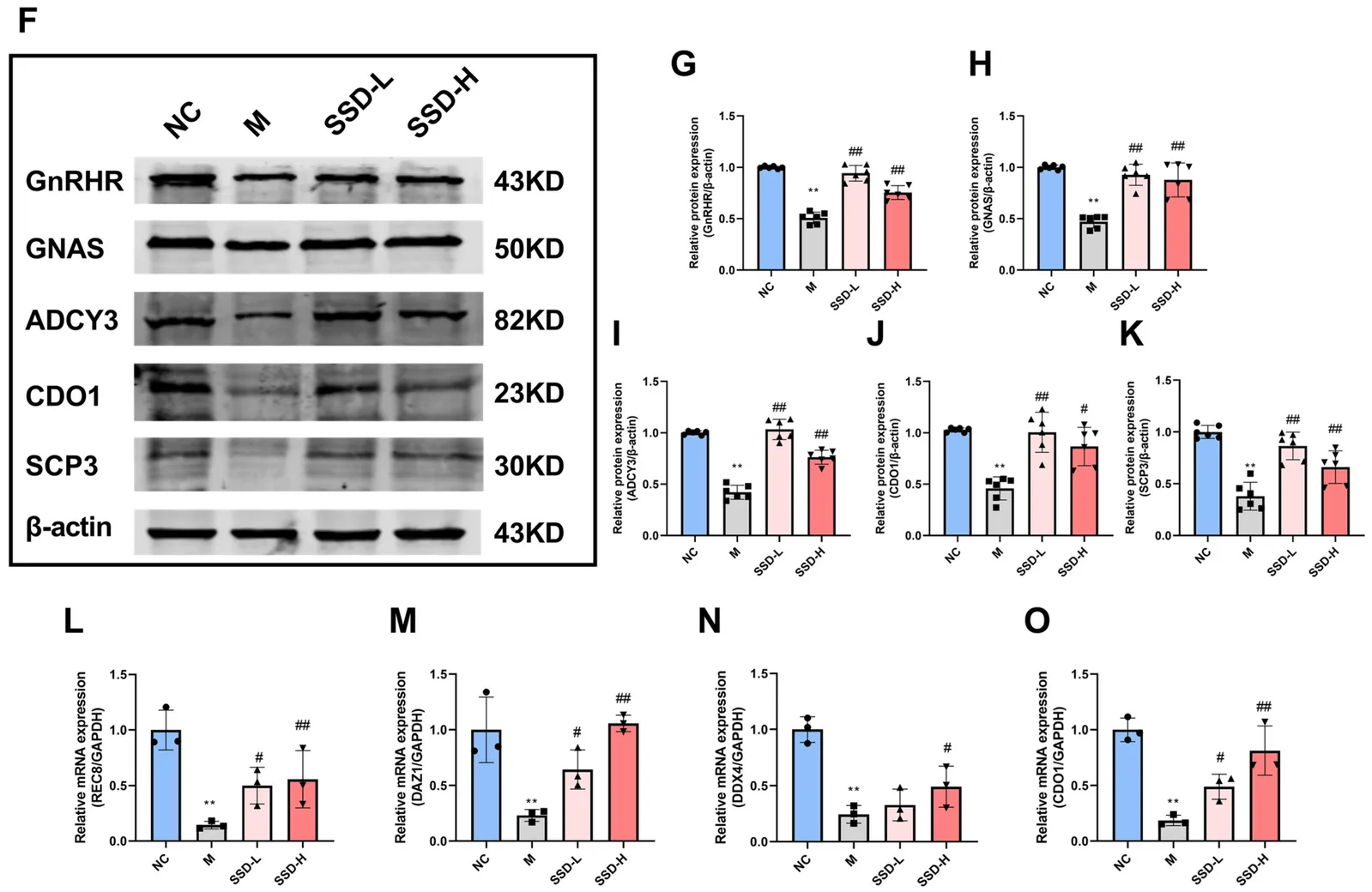
SSD modulates GnRHR signaling and taurine synthesis in GC-1 cells. (**A**–**E**) Protein levels of GnRHR, GNAS, SCP3, and CDO1 in GC-1 cells across treatment groups were detected using high-content imaging analysis. Scale bar = 100 μm. (**F**–**K**) Western blotting analysis of the protein expression levels of GnRHR, GNAS, ADCY3, SCP3, and CDO1 in GC-1 cells from each group. (**L**–**O**) qRT-PCR revealed SSd-induced upregulation of mRNA for meiosis-related genes REC8, DAZ1, DDX4 and CDO1. Data are presented as mean ± SD. ** *p* < 0.01 vs. NC group; ^#^ *p* < 0.05, ^##^ *p* < 0.01 vs. M group.

To further elucidate the effects of SSd on the GnRHR signaling pathway and taurine biosynthesis, high-content imaging analysis was performed, revealing that SSd markedly enhanced the expression of GnRHR, GNAS, SCP3, and CDO1 (Figure 8A–E). Western blotting corroborated these results by demonstrating elevated levels of GnRHR pathway and CDO1 proteins (Figure 8F–K). Consistently, qRT-PCR analysis showed that SSd significantly upregulated the mRNA expression levels of key meiosis-related genes (REC8, DAZ1, and DDX4) and CDO1 (Figure 8L–O).

## 3. Discussion

Over the past few decades, the global decline in male fertility has become a major public health concern, characterized by well-documented reductions in semen quality and quantity [29,30]. DOX, among the most extensively used chemotherapeutic drugs, exhibits potent anticancer efficacy and remains a mainstay of oncologic treatment regimens [31]. However, its clinical use is limited by severe toxicities, including cardiotoxicity, nephrotoxicity, hepatotoxicity, and reproductive toxicity [32,33,34,35,36]. Long-term chemotherapy with DOX induces gonadal toxicity, resulting in reduced testicular weight and decreased testosterone levels. Excessively high concentrations of ROS are generated during metabolism, which trigger DNA damage and ultimately impair spermatogenesis [37]. With the rising global incidence of cancer and increased reliance on chemotherapy, safeguarding male reproductive health has become a pressing clinical priority [38]. Most preclinical studies have evaluated chemical antioxidants or hormonal agents for chemotherapy-induced testicular injury, but adverse effects may limit their clinical use. The multi-component nature of traditional Chinese medicines has prompted interest in their potential reproductive-protective effects and provided the rationale for evaluating CH in this study.

In TCM, CH has long been recognized for its ability to soothe the liver, relieve stagnation, and regulate *qi* flow [25]. Spermatogenic dysfunction—manifesting as oligospermia, asthenospermia, or teratospermia—is traditionally attributed to syndromes such as liver *qi* stagnation, kidney essence deficiency, *qi* and blood insufficiency, or damp-heat accumulation [39]. The TCM principle of the “shared origin of the liver and kidney” is fundamental: the liver governs free coursing, while the kidney stores essence. When liver *qi* stagnates, it impairs the kidney’s capacity to consolidate essence, disrupting sperm production and release [40]. As a principal herb for soothing the liver, harmonizing the Shaoyang meridian, and uplifting yang *qi*, CH restores physiological *qi* dynamics and supports reproductive function. It is therefore a common component of formulas targeting infertility associated with liver *qi* stagnation and impaired *qi* flow. Corroborating its therapeutic relevance, Bupleurum was identified as the most frequently prescribed herb in formulas for acute orchitis [41].

GnRH is a pivotal neuropeptide that regulates the hypothalamic–pituitary–gonadal axis. Age-related declines in reproductive competence are largely attributable to reduced GnRH secretion. GnRH is a pivotal regulator of the hypothalamic–pituitary–gonadal axis. GnRH and GnRHR have also been detected in adult mammalian testes [42]. Our previous studies showed that CH increased the testicular index and GnRHR expression in rats [26]. In the present study, CH improved histological and spermatogenic indices and increased GnRHR and meiosis-related proteins in DOX-treated mice.

To clarify whether these effects are mediated via GnRH signaling, we administered cetrorelix, a GnRH antagonist widely used in assisted reproduction to suppress GnRHR expression [43]. Cetrorelix attenuated the CH-associated improvements in sperm motility and testosterone levels. This result supports the involvement of GnRHR signaling in the effects of CH extract in vivo.

The mitochondrial respiratory chain is a primary source of reactive oxygen species (ROS) [44]. Excess superoxide anions that escape neutralization by endogenous antioxidants or enzymes accumulate, leading to oxidative stress, mitochondrial dysfunction, and subsequent cell death [45]. Elevated ROS levels in testicular tissue induce oxidative damage that impairs spermatogenesis [46]. In our model, ROS levels were quantified by flow cytometry, while superoxide dismutase (SOD), malondialdehyde (MDA), and catalase (CAT) were measured using chemiluminescence assays. CH reduced testicular ROS and MDA levels and increased SOD and CAT activities. Cetrorelix attenuated these changes, suggesting that GnRHR signaling may contribute to the antioxidant effects of CH extract.

To further elucidate the underlying mechanisms, we performed metabolomic profiling to identify differential testicular metabolites between CH-treated and model groups. CH administration significantly increased testicular taurine levels, a finding corroborated by biochemical assay validation. We thus propose that CH enhances taurine biosynthesis. CH increased testicular taurine levels and the expression of CDO1 and FMO1, two enzymes associated with taurine biosynthesis [47,48]. Cetrorelix attenuated these increases. These parallel changes are consistent with enhanced taurine metabolism but do not establish direct GnRHR-to-enzyme regulation.

Recent studies suggest that taurine supports spermatogenesis [49,50], a process that depends on meiosis [51,52,53]. To further investigate this relationship, we analyzed the protein expression of SCP3 and REC8, two key regulators of meiosis, and quantified the mRNA levels of meiosis-related genes using quantitative real-time PCR (qRT-PCR). CH increased SCP3, REC8, and several meiosis-related transcripts, whereas cetrorelix attenuated these changes. The findings link the CH response to taurine-related and meiotic processes but do not establish that taurine directly mediates meiotic recovery.

Previous work in our laboratory revealed that activation of the GnRHR signaling pathway mitigates DOX-induced spermatogenic dysfunction [27,28]. To determine whether CH modulates this pathway, we examined GnRHR-related protein and mRNA expression using WB and RT-PCR. CH increased GnRHR-related protein and mRNA readouts, and cetrorelix attenuated several of these responses. These findings support a contribution of GnRHR signaling to the effects of CH extract but do not establish that this pathway is the primary or exclusive mechanism.

UHPLC–MS/MS detected saikosaponins A and D (SSD) in the CH extract. In preliminary GC-1 assays, SSD showed the strongest cytoprotective activity among the tested constituents and was selected for further evaluation. SSD reduced apoptosis, ROS accumulation, and inflammatory-gene expression, supporting its designation as a candidate bioactive constituent.

We employed high-content imaging and Western blotting to assess GnRHR activation, SCP3, and CDO1 expression. Both low and high doses of SSD enhanced GnRHR signaling pathway activity and increased SCP3 and CDO1 expression. qRT-PCR further confirmed elevated mRNA levels of meiosis-related genes following SSD treatment. Collectively, these results suggest that SSD is likely the key active compound underlying Bupleurum’s therapeutic efficacy in restoring spermatogenesis. SSD increased GnRHR-related, CDO1, and meiosis-related markers in GC-1 cells. However, cetrorelix or GnRHR knockdown was not used in vitro. These findings are therefore associative and do not establish that SSD acts directly through GnRHR. Further studies using receptor blockade or genetic manipulation are required to define the mechanism of SSD.

## 4. Materials and Methods

### 4.1. Chemicals and Drugs

DOX (Powerdone Pharmaceutics Co., Ltd. Datong, China; Batch No. 02231101). The Annexin V/propidium iodide (PI) apoptosis detection kit (BD Biosciences, Franklin Lakes, NJ, USA), and the fluorescent probe 2′,7′-dichlorodihydrofluorescein diacetate (DCFH-DA) (Elabscience Biotechnology, Beijing, China).

### 4.2. Preparation of Bupleurum Chinense Extract

CH was authenticated by Professor Suiqing Chen, Henan University of Chinese Medicine. To prepare the aqueous extract, 1 kg of crude CH was decocted in 10 L of distilled water, filtered through Whatman filter paper (GE Healthcare, Chicago, IL, USA), concentrated under reduced pressure, and lyophilized. The resulting powder was reconstituted in normal saline immediately before oral administration.

### 4.3. Animals and Ethical Statement

Male Kunming mice (weighing 25 ± 2 g) were provided by Charles River Laboratories (Beijing, China; License No. SCXK [Beijing] 2021-0011). Mice were housed under standard laboratory conditions (22 ± 2 °C, 12 h light/dark cycle) with ad libitum access to food and water. All experimental procedures were approved by the Institutional Animal Care and Use Committee (IACUC Approval No. IACUC-2025077046) and conducted in accordance with the Guide for the Care and Use of Laboratory Animals.

### 4.4. Experimental Design and Drug Administration

To assess CH’s pharmacological effects, mice were randomly assigned to six groups (*n* = 10/group): control, DOX-induced model (M), positive control (Shengjing pill, Y), CH low-dose (CH-L, 1.87 g/kg), CH medium-dose (CH-M, 3.75 g/kg), and CH high-dose (CH-H, 7.5 g/kg). Spermatogenic dysfunction was induced via a single intraperitoneal injection of DOX (30 mg/kg) in all groups except the control. Beginning on the day of modeling, treatment groups received daily oral administration of Y (suspension form; Wansheng Pharmaceutical Co., Ltd., Zunyi, China) or CH (decoction form; Beijing Tongrentang, Jiaozuo, China) for 14 consecutive days.

To further elucidate the role of GnRH signaling, mice were divided into six groups: control (NC), control + cetrorelix (NC + CE), DOX injury (M), DOX + cetrorelix (M + CE), CH, and CH + cetrorelix (CH + CE). Antagonist groups received daily intraperitoneal injections of CE (300 µg/kg) at 10:00 a.m., while the other groups received saline. At 6:00 p.m., the CH and CH + CE groups were administered CH (3.75 g/kg) by gavage; other groups received saline. The regimen was continued for 15 days. On day 14, all groups except the NC group received a single intraperitoneal injection of doxorubicin (30 mg/kg) at 18:00 to establish testicular injury. Administration was repeated on the final day.

### 4.5. Sample Collection and Processing

After the treatment period, mice were euthanized through an intraperitoneal injection of sodium pentobarbital (3%, 30 mg/kg). The epididymis was immediately dissected for sperm quality assessment as previously described [27,54]. Testicular tissues were either fixed in 4% paraformaldehyde for histological examination or flash-frozen at −80 °C for subsequent biochemical analyses.

### 4.6. Histopathological Evaluation

Following dissection, testes were fixed overnight in 4% paraformaldehyde at 4 °C, then dehydrated, cleared, and embedded in paraffin. Paraffin blocks were sectioned at 5 μm thickness, and four non-consecutive sections per testis were mounted on glass slides [28]. After hematoxylin and eosin (H&E) staining, histopathological changes were examined under a light microscope (NIKON Eclipse CI, Tokyo, Japan) for morphological assessments.

### 4.7. Hormone Measurement by ELISA

Serum concentrations of gonadotropin-releasing hormone (GnRH) and testosterone were determined using commercial ELISA kits (Elabscience Biotechnology, Beijing, China; catalog numbers E-EL-0071c for GnRH and E-EL-0155c for testosterone), strictly following the manufacturer’s instructions.

### 4.8. Evaluation of Oxidative Stress Markers

Testicular tissues were homogenized in ice-cold PBS and centrifuged at 15,000× *g* for 10 min at 4 °C. Total protein concentrations were measured using a bicinchoninic acid protein assay kit. SOD and CAT activities and MDA levels were quantified using commercial kits (Nanjing Jiancheng Bioengineering Institute, Nanjing, China).

### 4.9. Immunofluorescence Analysis

Testicular sections were deparaffinized, subjected to antigen retrieval, and blocked with BSA for 30 min. Sections were incubated with primary antibodies (1:100) for 16 h at 4 °C, washed five times with PBST, and incubated with Cy3-conjugated secondary antibodies for 1 h. After DAPI counterstaining, sections were mounted with antifade medium and imaged by microscopy.

### 4.10. Cell Culture and Treatment

The mouse spermatogonial cell line GC-1 was obtained from the American Type Culture Collection (ATCC, Manassas, VA, USA). Cells were cultured in high-glucose Dulbecco’s Modified Eagle Medium (DMEM) supplemented with 10% fetal bovine serum, 1% L-glutamine, and 1% penicillin–streptomycin, and maintained at 37 °C in a humidified 5% CO_2_ atmosphere.

Cells were passaged at >80% confluence. Cells were washed twice with PBS, digested with 1 mL trypsin, and the reaction was stopped by adding 1 mL culture medium. After gentle mixing, the suspension was aliquoted into 1.5 mL tubes and centrifuged at 1000 rpm for 5 min. Cells were finally seeded into culture dishes with complete medium and incubated at 37 °C in 5% CO_2_.

GC-1 cells were seeded in 96-well plates or culture dishes at 4 × 10^4^ cells/mL. The groups were NC, DOX (0.5 μM), DOX + SSD-L (0.5 μM DOX + 0.1 μM SSD), and DOX + SSD-H (0.5 μM DOX + 1 μM SSD). Treatment was administered simultaneously with model establishment.

### 4.11. Assessments of Apoptosis, Oxidative Stress, and Cell Viability

Apoptosis was analyzed using Annexin V-FITC/propidium iodide (PI) staining followed by flow cytometry. Briefly, 500 μL 1 × Binding buffer, 5 μL Annexin V-FITC and 5 μL PI were added to each sample, and samples were detected by flow cytometry (BD FACSAria III, Newark, NJ, USA). For ROS detection, 1 μL positive reagent and 1 mL DMEM were added to the positive control tube. For the negative control tube, cells were incubated with DMEM containing DCFH-DA diluted at a volume ratio of 1000:1. Cell viability was determined with the CCK-8 assay (Abcam, Cambridge, UK), and absorbance was measured at 450 nm using a microplate reader. All procedures were conducted as previously described [27,54].

### 4.12. RNA Extraction and Quantitative Real-Time PCR (qRT-PCR)

Total RNA was extracted from testicular tissues or GC-1 cells using a commercial kit (Solarbio, Beijing, China; R1200). RNA purity was assessed using the A260/A280 ratio. Data were analyzed using the 2^−ΔΔCt^ method. Reverse transcription was performed with the HiScript^®^ II 1st Strand cDNA Synthesis Kit (Vazyme Biotech, Nanjing, China), and quantitative PCR (qPCR) was conducted using ChamQ™ Universal SYBR^®^ qPCR Master Mix (Vazyme Biotech). Primer sequences are listed (Supplementary Table S2).

### 4.13. Western Blot Analysis

Proteins were extracted from testicular tissues or GC-1 cells using a cytoplasmic and membrane protein extraction kit (Applygen Technologies, Beijing, China). GnRHR and GNAS were analyzed in membrane fractions, whereas all other target proteins were analyzed in cytoplasmic fractions. Primary-antibody dilutions and sources are listed in Supplementary Table S3. Protein bands were visualized using the Odyssey infrared imaging system and quantified using Image Studio software (LI-COR, Version 5.2.5). β-Actin served as the loading control.

### 4.14. High-Content Imaging Analysis

GC-1 cells were seeded in 96-well black plates with 200 μL of complete culture medium per well and incubated overnight in an incubator. After drug treatment, the cells were cultured for another 24 h. The culture medium was aspirated, and cells were fixed with 4% paraformaldehyde at room temperature for 30 min. Following removal of the fixative, cells were rinsed once with PBS, permeabilized with 0.5% Triton X-100 for 20 min at room temperature, and then blocked with blocking solution for 30 min.

After discarding the blocking solution, the corresponding primary antibodies were added to each well, and the plates were incubated overnight at 4 °C. On the next day, wells were washed five times with PBS. Cells were then incubated with fluorescent secondary antibodies for 1 h at room temperature in the dark, followed by nuclear counterstaining with 4′,6-diamidino-2-phenylindole (DAPI) for 10 min. After final PBS washing, protein expression was detected using a high-content imaging system.

### 4.15. Statistical Analysis

Data are expressed as mean ± *SD* and analyzed using SPSS 19.0 (IBM, Armonk, NY, USA). Intergroup differences were assessed by one-way ANOVA followed by [verified post hoc test]. A two-sided *p* < 0.05 was considered statistically significant.

## 5. Conclusions

Overall, the CH extract appears to mitigate spermatogenic dysfunction by regulating the GnRHR signaling pathway and promoting taurine synthesis.

## Data Availability

The original contributions presented in this study are included in the article. Further inquiries can be directed to the corresponding author.

## Supplementary Materials

The following supporting information can be downloaded at: https://www.mdpi.com/article/10.3390/ph19081280/s1, Figure S1. Effects of CH on Hepatic and Renal Function Indicators (A) Serum AST levels; (B) Serum aALT levels; (C) Serum BUN levels; (D) Serum Cr levels. Data. Figure S2. CH on HPLC/MS. Table S1. LC-MS Analysis of Bupleurum Extract. Figure S3. Testicular Metabolomics Reveals the Ameliorative Effect of Bupleurum on Spermatogenic Dysfunction. (A) Principal component analysis (PCA) score plot; (B) PCA plots comparing: (i) Normal vs. Model groups, and (ii) Model vs. CH groups; (C) Orthogonal partial least squares-discriminant analysis (OPLS-DA) score plots comparing: (i) Normal vs. Model groups, and (ii) Model vs. Bupleurum-treated (CH) groups; (D) OPLS-DA S-plots comparing: (i) Normal vs. Model groups, and (ii) Model vs. CH groups; (E) Heatmap of differential metabolites among Normal, Model, and CH groups. Figure S4. CH promotes taurine biosynthesis in the testes. Figure S5. Screening of Saikosaponin Activity Using a Doxorubicin-Injured GC-1 Cell Model. Table S2. Primer sequences used in the study. Table S3. Dilutions and sources of primer antibodies.

## Abbreviations

The following abbreviations are used in this manuscript:

ATCC	American Type Culture Collection
CH	*Radix Bupleuri Chinensis/*Chaihu
DMEM	Dulbecco’s Modified Eagle Medium
GnRHR	GnRH receptor
H&E	Hematoxylin and eosin
IACUC	Institutional Animal Care and Use Committee
PBS	Phosphate-buffered saline
PCA	Principal component analysis
PI	Propidium iodide
ROS	Reactive oxygen species
SOD	Superoxide dismutase
STAR	Steroidogenic acute regulatory protein
SSD	Saikosaponin D
TCM	Traditional Chinese Medicine
WB	Western blotting
