# Supplementary material for: Radix Bupleuri Chinensis Extract Ameliorates Doxorubicin-Induced Spermatogenic Dysfunction: Involvement of the GnRHR Signaling Pathway and Taurine Biosynthesis
Source: Pharmaceuticals (Basel). 2026 Aug 13;19(8):1280. doi: 10.3390/ph19081280 (PMC13516573; doi:10.3390/ph19081280)
Supplement: Supplementary file 1 [file pharmaceuticals-19-01280-s001.zip › pharmaceuticals-4470449-supplementary.pdf]

1. **Effects of CH on Hepatic and Renal Function Indicators**

This study aimed to explore whether high-dose CH (CH-H) induces hepatotoxicity and nephrotoxicity. Kunming (KM) mice were divided into two groups: the normal control (NC) group was given an equal volume of pure water via intragastric administration, whereas the NC+CH-H (7.5 g/kg) group received high-dose aqueous decoction of CH alone by gavage. After 30 consecutive days of intervention, mouse serum was collected for the measurement of serum aspartate aminotransferase (AST), alanine aminotransferase (ALT), blood urea nitrogen (BUN), and creatinine (Cr). The detection results revealed that no significant differences were observed in the four indicators (AST, ALT, BUN and Cr) between mice treated with high-dose CH and those in the normal control group. Collectively, these results verified that high-dose CH does not exert hepatotoxic or nephrotoxic effects.

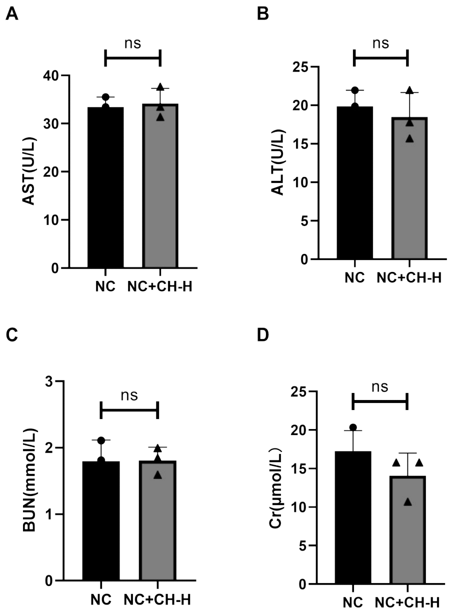

Fig. S1. Effects of CH on Hepatic and Renal Function Indicators

(A) Serum AST levels (B) Serum aALT levels (C) Serum BUN levels(D) Serum Cr levels. Data presented as mean ± *S.D.* for three independent experiments. ^{ns} p > 0.05 vs. NC group.

2. LC-MS Analysis of Bupleurum Extract

Chemical constituents in the CH aqueous extract were analyzed using liquid chromatography-mass spectrometry (LC-MS). Key identified saikosaponins included Saikosaponin A

(SSA, Peak number 2), Saikosaponin B_1 (SSB_1, Peak number 3), Saikosaponin B_2 (SSB_2, Peak number 2), Saikosaponin C (SSC, Peak number 1), and Saikosaponin D (SSD, Peak number 4) (Fig. S2). Relevant information can be found in Supplementary Table S1.

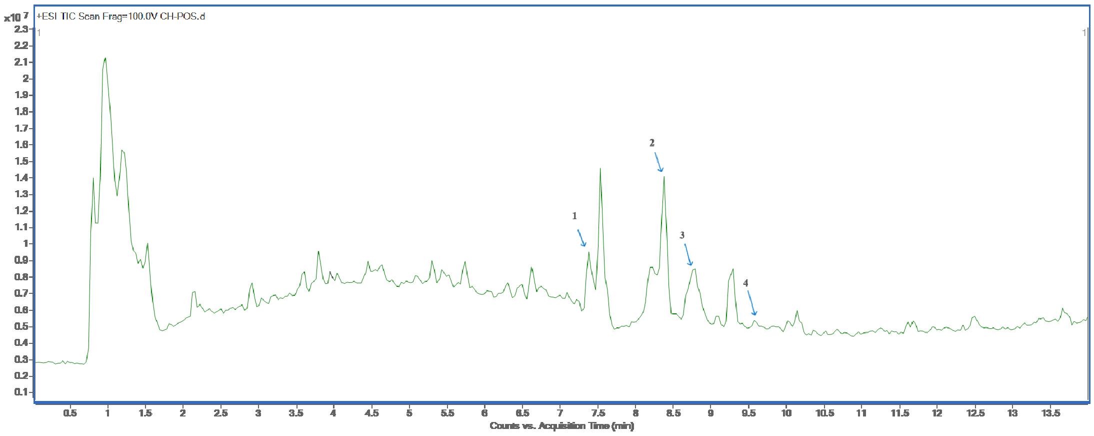

Fig. S2. CH on HPLC/MS. Chromatographic separation with Acclaim™ RSLC 120 C18 column (2.1×100 mm，1.6 μm；Thermo Scientific). Column temperature: 30 °C ; Volume flow rate: 0.3 mL / min; Injection volume: 2 μL; Mobile phase: (A)0.1 % formic acid - (B) acetonitrile; Gradient elution program: 0 ~ 5 min, 5 ~ 35 % B; 5-14 min, 35-95 % B; 14-15 min, 95-5 % B. Detection wavelength 254nm. Electrospray ion source (ESI), positive and negative ion detection modes; Ion source energy 3.0 eV; Collision energy 8.0 eV; Mass scan range m/z 50-1500; Capillary voltages: 3500 V (ESI +) and 3200 V (ESI +), respectively; Atomizing air pressure: 2 Bar ; Desolvation gas temperature: 230 °C and flow rate: 8 mL/min.

Table S1. LC-MS Analysis of Bupleurum Extract.

Peak number	Compound	RT/min	Molecular formula	m/z Positive ion	Ppm	Ion fragment	M+
1*	Saikosaponin C	7.405	$C_{48}H_{78}O_{17}$	949.5137	0.6319	439.3574[M-$C_{18}H_{32}O_{15}$]$^+$<br>421.3467[M-$C_{18}H_{32}O_{15}$-H_2O]$^+$<br>403.3374[M-$C_{18}H_{32}O_{15}$-H_2O×2]$^+$	[M+Na]$^+$
2*	Saikosaponin A/B2	8.346	$C_{42}H_{68}O_{13}$	803.4592	4.9785	763.4671[M+H-H_2O]$^+$<br>745.4576[M+H-H_2O×2]$^+$<br>455.3602[M+H-fuc-glc-H_2O]$^+$<br>437.3496[M+H-fuc-glc-H_2O×2]$^+$<br>419.3396[M+H-fuc-glc-H_2O×3]$^+$<br>407.3394[M+H-fuc-glc-H_2O×2-CH_2O]$^+$	[M+Na]$^+$
3*	Saikosaponin B1	8.785	$C_{42}H_{68}O_{13}$	803.4540	-1.4935	455.3518[M+H-fuc-glc-H_2O]$^+$<br>437.3411[M+H-fuc-glc-H_2O×2]$^+$	[M+Na]$^+$
4*	Saikosaponin D	9.665	$C_{42}H_{68}O_{13}$	803.4546	-0.7468	455.3517[M+H-fuc-glc-H_2O]$^+$<br>437.3412[M+H-fuc-glc-H_2O×2]$^+$	[M+Na]$^+$

3. Testicular metabolomic analysis of Bupleurum's effect on ameliorating spermatogenic dysfunction

To further elucidate the mechanism by which CH ameliorates spermatogenic dysfunction, we performed metabolomic profiling of testicular tissue to identify reliably altered metabolites.

This analysis revealed that CH significantly elevated testicular taurine levels (**Fig. S3.**). Given taurine's critical role in spermatogenesis, we subsequently examined the expression of its key biosynthetic enzymes, CDO1 and FMO1.

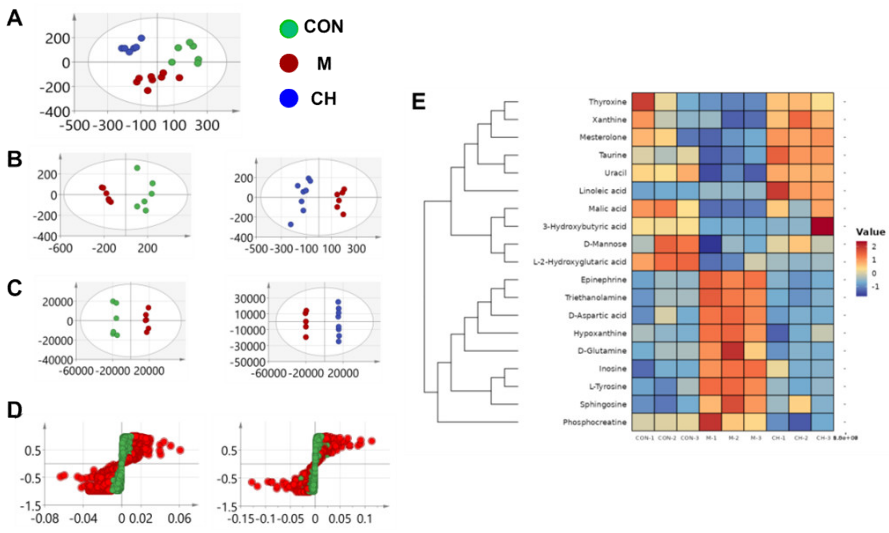

Fig. S3. Testicular Metabolomics Reveals the Ameliorative Effect of Bupleurum on Spermatogenic Dysfunction.

(A) Principal component analysis (PCA) score plot. (B) PCA plots comparing: (i) Normal vs. Model groups, and (ii) Model vs. CH groups. (C) Orthogonal partial least squares-discriminant analysis (OPLS-DA) score plots comparing: (i) Normal vs. Model groups, and (ii) Model vs. Bupleurum-treated (CH) groups. (D) OPLS-DA S-plots comparing: (i) Normal vs. Model groups, and (ii) Model vs. CH groups. (E) Heatmap of differential metabolites among Normal, Model, and CH groups.

4. CH promotes taurine biosynthesis in the testes.

Taurine levels in testicular tissue were measured using a taurine assay kit. CH significantly increased testicular taurine content ($p < 0.01$), an effect abolished by co-administration of the GnRH antagonist cetrorelix.

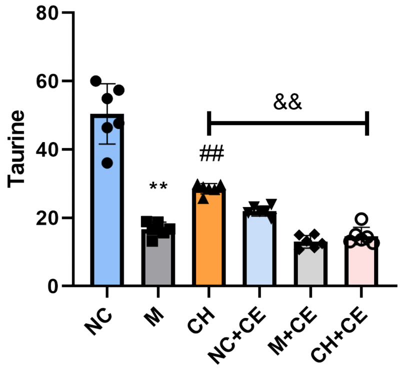

Fig. S4. CH promotes taurine biosynthesis in the testes.

Data presented as mean ± *S.D.* for three independent experiments. $^{**}p < 0.01$ vs. NC group; $^{\#\#}p < 0.01$ vs. M group.

5. Screening of Saikosaponin Activity Using a Doxorubicin-Injured GC-1 Cell Model

To identify the active components in Bupleurum responsible for ameliorating spermatogenic dysfunction, we screened the bioactivity of several saikosaponins—including saikosaponin A (SSA), B1 (SSB1), B2 (SSB2), B4 (SSB4), C (SSC), and D (SSD)—using a doxorubicin-injured GC-1 cell model. Among these, SSd exhibited the most pronounced protective effect, and was therefore selected for subsequent experiments.

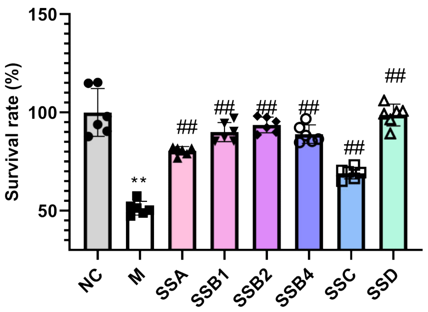

Fig. S5. Screening of Saikosaponin Activity Using a Doxorubicin-Injured GC-1 Cell Model.

Data presented as mean ± *S.D.* for three independent experiments. $^{**}p < 0.01$ vs. NC group; $^{\#\#}p < 0.01$ vs. M group.

Table S2. Primer sequences used in the study.

Target	Forward primer (5′–3′)	Reverse primer (5′–3′)	Gene ID
GnRHR	TGCTCGGCCATCAACAACA	GGCAGTAGAGAGTAGGAAAAGGA	14715
GNAS	CAGAGCCTCCATTGGGGTC	GCTTCTCGCTCAACTGGGG	14683
PKA	AGGGCAGGACATGGACATTG	CGCCTTATTGTAACCCTTGCTG	18749
DDX4	GAGGGGGAAGAGGCAGTTTC	TGGTAAGTGTCACCATTGCCT	13206
DAZ1	ATACCTCCGGCTTATACAACTGT	GACTTCTTTTGCGGGCCATTT	13164
SCP3	AGCCAGTAACCAGAAAATTGAGC	CCACTGCTGCAACACATTCATA	20962
REC8	GGTCATCACCTTACAGGAGGC	TCTGCGATCAGCAGTTCTAAGT	56739
SMC1B	CATGAGGGAAAACGTCAGCAG	TGACACAGATCAAGCAGTCTTC	140557
STAR	CGGGTGGATGGGTCAAGTTC	GCACTTCGTCCCCGTTCTC	20845
CDO1	GGGGACGAAGTCAACGTGG	ACCTGTATTGATCGAATTTGGCA	12583
GAPDH	TGTGTCCGTCGTGGATCTGA	TTGCTGTTGAAGTCGCAGGAG	14433

Table S3. Dilutions and sources of primer antibodies.

Target	Dilution	Source
GnRHR	1:500	19950-1-AP; Proteintech
GNAS	1:1000	ab283266; Abcam
PKA	1:1000	12232-1-AP; Proteintech
ADCY3	1:1000	19492-1-AP; Proteintech
p-CREB	1:1000	9198; Cell Signaling Technology
CREB	1:1000	12208-1-AP; Proteintech
CDO1	1:1000	12589-1-AP; Proteintech
FMO1	1:1000	12589-1-AP; Proteintech
SCP3	1:1000	23024-1-AP; Proteintech
REC8	1:1000	ab192241; Abcam
β-Actin	1:5000	66009-1-Ig; Proteintech
